# Correlating *In Vitro* Splice Switching Activity With Systemic *In Vivo* Delivery Using Novel ZEN-modified Oligonucleotides

**DOI:** 10.1038/mtna.2014.63

**Published:** 2014-11-25

**Authors:** Suzan M Hammond, Graham McClorey, Joel Z Nordin, Caroline Godfrey, Sofia Stenler, Kim A Lennox, CI Edvard Smith, Ashley M Jacobi, Miguel A Varela, Yi Lee, Mark A Behlke, Matthew J A Wood, Samir E L Andaloussi

**Affiliations:** 1 Department of Physiology, Anatomy and Genetics, University of Oxford, Oxford, UK; 2 Department of Laboratory Medicine, Karolinska Institutet, Karolinska University Hospital Huddinge, Huddinge, Sweden; 3 Integrated DNA Technologies, Inc, Coralville, Iowa, USA

**Keywords:** antisense oligonucleotides, Duchenne muscular dystrophy, splice switching, ZEN modification

## Abstract

Splice switching oligonucleotides (SSOs) induce alternative splicing of pre-mRNA and typically employ chemical modifications to increase nuclease resistance and binding affinity to target pre-mRNA. Here we describe a new SSO non-base modifier (a naphthyl-azo group, “ZEN™”) to direct exon exclusion in mutant dystrophin pre-mRNA to generate functional dystrophin protein. The ZEN modifier is placed near the ends of a 2′-*O*-methyl (2′OMe) oligonucleotide, increasing melting temperature and potency over unmodified 2′OMe oligonucleotides. In cultured H2K cells, a ZEN-modified 2′OMe phosphorothioate (PS) oligonucleotide delivered by lipid transfection greatly enhanced dystrophin exon skipping over the same 2′OMePS SSO lacking ZEN. However, when tested using free gymnotic uptake *in vitro* and following systemic delivery *in vivo* in dystrophin deficient *mdx* mice, the same ZEN-modified SSO failed to enhance potency. Importantly, we show for the first time that *in vivo* activity of anionic SSOs is modelled *in vitro* only when using gymnotic delivery. ZEN is thus a novel modifier that enhances activity of SSOs *in vitro* but will require improved delivery methods before its *in vivo* clinical potential can be realized.

## Introduction

It has been estimated that nearly one tenth of all disease-causing mutations are caused by single-base pair substitutions affecting pre-mRNA splicing.^1^ Many of these mutations are amenable to splice switching therapy whereby small nucleic acids are directed toward *cis* splicing elements within pre-mRNA.^2,3^ These splice switching oligonucleotides (SSOs) can act through targeted binding to pre-mRNA to promote or inhibit recruitment of splicing factors so as to induce exon inclusion or exclusion, block pseudoexons from recognition, and influence alternative splicing.^2,4,5,6^ SSOs can be directed to target the disease-causing mutations directly, such as to block cryptic splicing in β-thalassemia, to promote inclusion of exons or to induce removal of exons containing premature termination codons. Alternately, SSOs can target regions around a mutation, such as for Duchenne muscular dystrophy (DMD), whereby frame-shift deletions in the *DMD* gene can be bypassed by removing additional exons to create an in-frame deletion that produces a partially functional, internally truncated dystrophin protein.^7,8^ It is the unique structure and function of dystrophin, with its large series of structural repeats in the central rod domain that allows this splice switching strategy to be successful.^4,9,10,11,12^

Two SSO chemistries have been utilized for DMD clinical studies; phosphorodiamidate morpholino (PMO) or 2′-*O*-methyl phosphorothioate RNA (2′OMePS RNA). These so-called second-generation oligonucleotide (ON) analogs increase target affinity and confer nuclease resistance. The first splice switching clinical trials for DMD utilizing a 2′OMePS SSO, called drisapersen,^13^ and a PMO SSO, eteplirsen,^14^ resulted in limited success. While these compounds are well tolerated, results of a phase 3 clinical trial (DMD114044) for drisapersen announced recently indicated that the trial had failed to meet its primary endpoint of demonstrating improvement over placebo (Prosensa update 19 December 2013). Eteplirsen has achieved better success; patients treated up to 2 years appear to have stabilized muscle function as seen by stability in their ability to perform the 6-minute walk test.^14^ However, for this approach to be successful, there remains an important need to develop new and modified forms of ONs that improve clinical efficacy.

To improve splice-switching efficacy, we exploited a novel non-base modifier which can be introduced to any anionic ON to enhance its binding affinity by increasing the *T*_m_. The modifier, N,N-diethyl-4-(4-nitronaphthalen-1-ylazo)-phenylamine (dubbed “ZEN™”), has very recently been successfully utilized in anti-microRNA ON (antagomir) designs, significantly improving the potency of 2′OMe-based antagomirs *in vitro.*^15^ Here, upon inclusion of ZEN into conventional 20-mer 2′OMePS RNA-based SSOs targeting exon 23 in *mdx* myotubes, the potency of the SSO was dramatically increased when using cationic lipid-mediated transfection. Inclusion of ZEN also demonstrated successful splice-switching activity with shorter 17-mer SSO sequences, which was absent in SSOs lacking the ZEN modifier. Surprisingly, even though well tolerated *in vivo*, ZEN modification conferred no advantage on the activity of SSOs following naked IV administration in *mdx* mice. This effect was replicated in gymnotic delivery experiments, suggesting that this modifier may hinder the efficiency of uptake of naked SSOs and is therefore more useful when combined with facilitated delivery.

## Results

### Design of ZEN-modified SSOs

**Figure 1:**
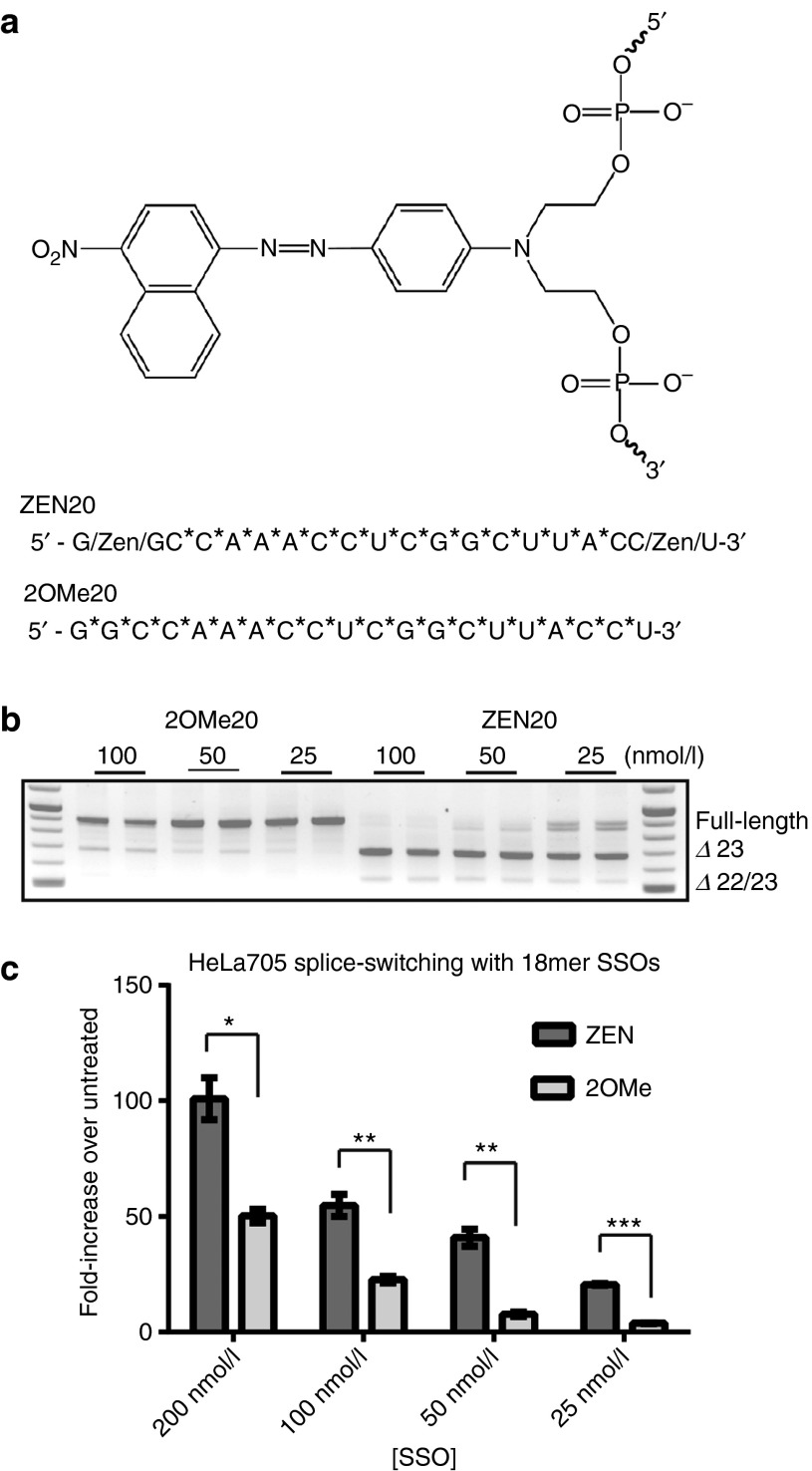
**ZEN-modified splice switching oligonucleotides (SSOs) are more potent *in vitro* than unmodified 2′OMePS following lipofection**. (**a**) Chemical structure of the modifier, N,N-diethyl-4-(4-nitronaphthalen-1-ylazo)-phenylamine “ZEN”. Sequences of two studied 20-mer 2′OMe sequences (Ex23 +2,-18) with and without ZEN modification, ZEN20 and 2OMe20 respectively, used for exon skipping in *mdx* cells. ZEN is located between the final and penultimate end nucleotides. *PS modification (**b**) A representative reverse transcription-polymerase chain reaction (RT-PCR) analysis of differentiated H2K *mdx* muscle cells lipofected with indicated SSOs. Derived from the *mdx* mouse model for Duchenne muscular dystrophy, these cells contain a stop codon within exon 23. Both 2OMe20 and ZEN20 are able to promote exon skipping in a dose dependent manner to generate Δ exon 23 transcripts, with the latter being substantially more potent. Exon 22+23 skipped transcript is regularly observed in *mdx* cells treated with high concentrations of a particularly active SSO.^45^ The double band present at the full-length position is due to a heterologous product caused by partial annealing of the skipped product with full-length product. (**c**) ZEN substantially improves splice switching in the splicing reporter system pLuc705 HeLa cells. 18-mer 2′OMePS SSO with and without ZEN modification was transfected at 25, 50, 100, and 200 nmol/l concentrations using LF2000. At all tested concentrations, ZEN outperforms the parent unmodified SSO. Luciferase expression was normalized to untreated pLuc705 HeLa cells and presented as fold-increase in luciferase expression over untreated cells. Experiments were performed at least three times in duplicate, with data showing the standard deviation. *P ≤ 0.05, **P ≤ 0.005, ***P ≤ 0.0005 (Student's *t*-tests).

2′OMe RNA is a widely used chemistry since it is a naturally occurring nucleic acid residue, which has no inherent chemical toxicity and is not known to trigger innate immune responses. When hybridized to an RNA target, 2′OMe ONs show increased binding affinity relative to DNA or RNA.^16^ However, due to its susceptibility to exonuclease digestion, PS internucleotide linkages are typically incorporated to stabilize against exonucleases.^17^ 2′OMePS RNAs have been successfully used for DMD exon skipping therapy both in *mdx* mice and in DMD patients.^18,19^ However, high doses are generally needed both *in vitro* and *in vivo* to obtain significant biological effects^20^ and a recent phase 3 clinical trial suggests that improvements in ON efficacy will be needed to demonstrate benefit to patients. While 2′OMe RNA ONs demonstrate enhanced binding affinity to RNA targets, some of this increase is lost with the addition of the PS internucleotide modification.^21,22^ Thus, we hypothesized that 2′OMePS SSOs would exhibit improved activity if a *T*_m_-enhancing modification such as ZEN was introduced, as previously reported for antagomirs.^15^ Incorporation of ZEN into any position within a DNA duplex increases *T*_m_ although the greatest effect on *T*_m_ was observed when ZEN was placed at the penultimate ends. In keeping with the incorporation pattern previously established in antagomirs, two ZEN moieties were inserted between the last and penultimate nucleotides on each end of the SSO (**Figure 1a**). Addition of the ZEN modification in this pattern for a 2′OMePS RNA raises *T*_*m*_ by ~4 °C when binding to an RNA target.^15^ A full list of sequences tested in this study is shown in **Table 1**.

### ZEN increases the potency of conventional 2′OMePS RNA-based SSOs

In order to assess the impact of ZEN on SSO-mediated exon skipping, we used a commonly studied 20-mer sequence (Ex23D +2–18) as the basis for comparison.^23^ Differentiated H2K *mdx* mouse muscle cells were transfected either with the parent 20-mer 2′OMePS RNA sequence (depicted 2OMe20) or an identical SSO having two ZEN modifications (denoted ZEN20) by cationic lipofection (**Figure 1a**). As seen in **Figure 1b**, both SSOs promote dose-dependent skipping of exon 23 in H2K *mdx* cells, however, addition of the ZEN modifier greatly increases the potency. At 25 nmol/l concentration, the relative skipping efficacy observed with ZEN20 surpasses the levels detected with 2OMe20 at fourfold higher concentrations (**Figure 1b**). In order to substantiate these findings and exclude the possibility that the observed improvement with ZEN was exclusive for Ex23D(+2–18) ON, we used an unrelated 18-mer sequence in another splicing reporter cell system, namely HeLa pLuc705 cells.^24^ Here, a stably transfected plasmid carrying the luciferase coding sequence is interrupted by an insertion of intron 2 from human β-globin pre-mRNA carrying a single point mutation which creates a 5′ splice site and activates a cryptic 3′ splice sites such that the pre-mRNA of luciferase retains a portion of intron 2 and will be incorrectly processed. Masking of the aberrant 5′splice site with an SSO redirects the splicing machinery and consequently functional luciferase is produced. Lipofection with the 18-mer 2′OMePS RNA promoted a dose-dependent increase in luciferase expression, with activity increased significantly upon addition of ZEN; reaching similar luciferase levels using 25 nmol/l ZEN modified SSO as compared to using 100 nmol/l concentration of the parent SSO (**Figure 1c**). Previous work has determined that the ZEN modification does not influence transfection efficiency.^15^ Therefore, these results suggest that the activity of conventionally used 2′OMePS RNA can be enhanced significantly by incorporating novel non-nucleotide modifiers that raise the overall *T*_m_ of SSOs.

### Impact of SSO length on exon skipping activity

**Figure 2:**
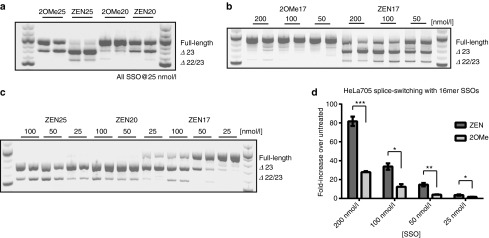
**Impact of splice switching oligonucleotides (SSO) length on splice switching activity.** (**a–c**) Representative reverse transcription-polymerase chain reaction (RT-PCRs) from H2K *mdx* cells transfected with LF2000. (**a**) The activity of 25-mer (2OMe25 and ZEN25) and 20-mer (2OMe20 and ZEN20) sequence lengths were compared with and without ZEN modification. The 25-mer SSOs improved skipping over the corresponding 20-mer sequences. Furthermore, in both cases, ZEN modified SSOs showed improved activity over unmodified 2′OMePS SSOs. (**b**) ZEN modified SSOs improve previously inactive 2′OMePS 17-mer (2OMe17). 2OMe17 (+2,-15) shows no activity up to 200 nmol/l concentrations. The addition of ZEN modifier rescued the activity to generate significant amounts of Δ exon 23 skipping. (**c**) Side by side dosing comparison of ZEN modified SSOs at 25-mer, 20-mer and 17-mer lengths. The longer the SSOs the better activity is observed in a dose dependent manner. (**d**) Luciferase activity increase in pLuc705 HeLa cell line transfected with 16-mer SSOs by lipofection. The ZEN modified SSO greatly improved the potency over the unmodified 2OMe16. Luciferase experiments were performed at least three times in duplicate, showing the standard deviation. *P ≤ 0.05, **P ≤ 0.005, ***P ≤ 0.0005 (Student's *t*-tests).

We next set out to validate how the length of SSOs impacts activity. Longer sequences have naturally higher overall *T*_m_ and typically induce increased splice switching activity. However, caution must be taken for high binding affinity ONs as they can also decrease specificity through binding at mismatched sites. When comparing the activity of DMD 2OMe25 and 2OMe20, with or without ZEN, the 25-mers displayed higher exon skipping activity at 25 nmol/l concentrations (**Figure 2a**). Again, the ZEN versions showed enhanced potency. Based on this, we tested a new set of shorter SSOs in order to determine the minimal length required for efficient exon skipping. In initial lipofection dose–response experiments, we tested a 17-mer sequence (-2,-18) based on 2′OMePS RNA with or without the ZEN modification. Strikingly, we were unable to detect any Ex23 skipping with the 2OMe17 whereas ZEN17 promoted robust skipping at all tested concentrations (**Figure 2b**), although was less potent than ZEN20 and ZEN25 (**Figure 2c**). These findings were substantiated in independent experiments in the HeLa pLuc705 model, where a 16-mer ZEN-based sequence showed similar activity to the previously tested 18-mer 2′OMePS RNA (**Figure 1c**) and significantly exceeded the potency of a corresponding 16-mer devoid of ZEN (**Figure 2d**). Thus, incorporation of ZEN allows for the use of shorter SSOs that could improve bioavailability *in vivo* similar to what has been previously reported for LNA-based antagomirs and SSOs as well as gapmers based on tricycloDNA.^25,26,27^

### Specificity and cytotoxicity profile of ZEN-based SSOs

**Figure 3:**
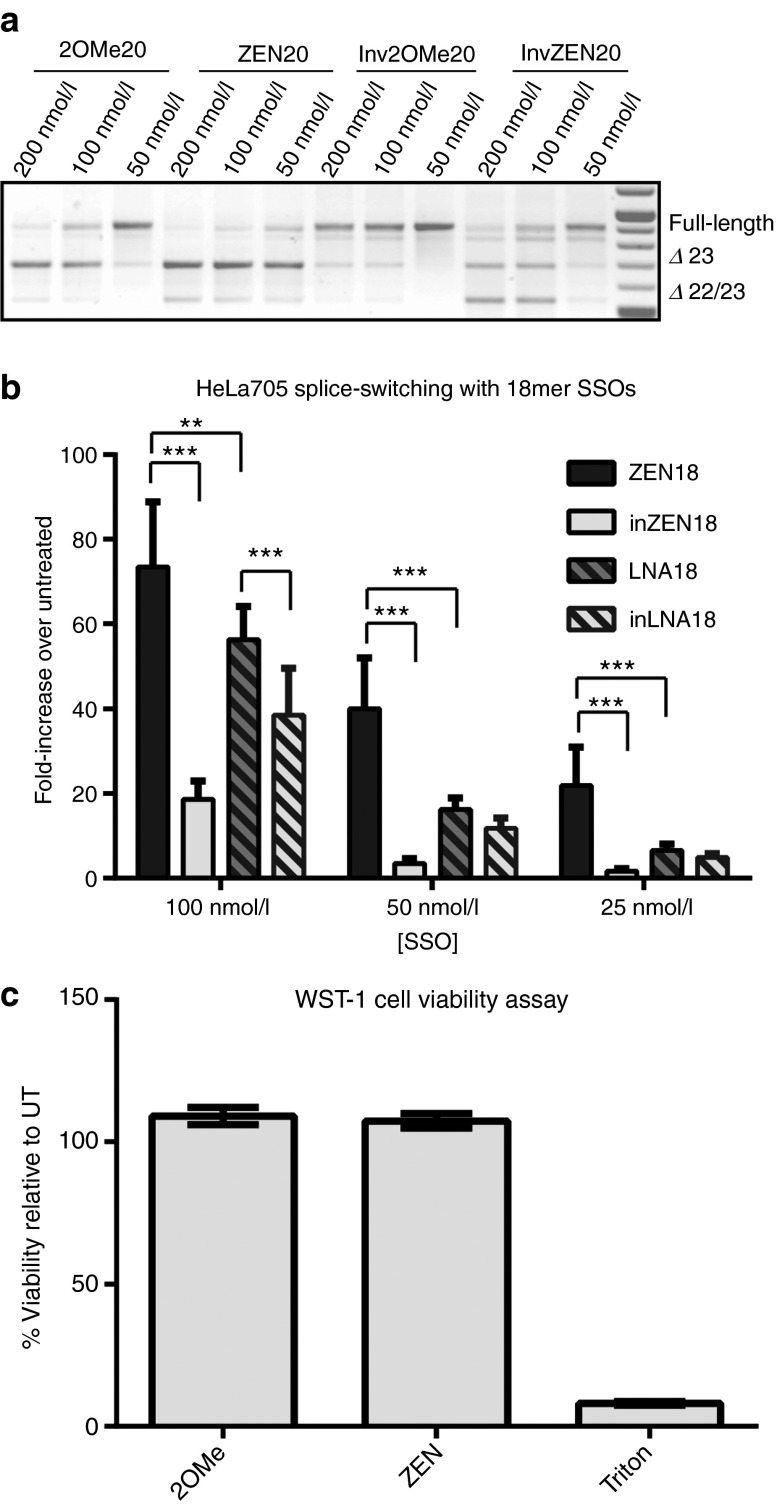
**Effect of ZEN modification on specificity and cytotoxicity.** (**a**) Reverse transcription-polymerase chain reaction (RT-PCR) of H2K *mdx* cells following lipofection of 20-mer splice switching oligonucleotides (SSOs). SSOs 2OMe20 and ZEN20 were compared to similar version with four central bases inverted (inv2OMe20 and invZEN20) to assess target sequence specificity. Inverting the four bases to introduce four mismatches to the dystrophin pre-mRNA resulted in a drop in levels of exon 23 skipping for both chemistries. The invZEN20 retained some of the activity though the relative reduction in activity appears similar for both chemistries compared to their parent sequences. (**b**) Luciferase activity in pLuc705 HeLa cell line transfected with 18-mer SSOs by lipofection. ZEN18 showed significantly improved activity over the mismatch invZEN18mer containing a four base inversion. In contrast LNA18 SSO was unable to show significantly improved activity over mismatched invLNA18, containing a four base inversion, at 50 and 25 nmol/l concentrations. (**c**) Wst-1 cell proliferation assay as a measurement of mitochondrial dehydrogenase activity. Cells transfected with 200 nmol/l of either 2OMe20 or ZEN20 had no effect on cell viability at 24 hours post-transfection. Triton was used as a positive control. Experiment was performed twice in quadruplicate. *P ≤ 0.05, **P ≤ 0.005, ***P ≤ 0.0005(Student's *t*-tests); UT, untreated.

In light of the high potency observed for the ZEN-modified SSOs, we sought to investigate whether use of a *T*_m_-raising modification impairs the specificity of SSOs or imparts increased toxicity. To address this, we used the initial 20-mer sequences (2OMe20 and ZEN20) and compared them with versions having four central bases inverted (inv2OMe20 and invZEN20). In keeping with the previous data, both 2OMe20 and ZEN20 dose-dependently induced robust exon skipping with the latter SSO being more active (**Figure 3a**, one representative experiment of three). As expected, by inverting the four bases and thereby introducing four mismatches to the dystrophin pre-mRNA a drop in levels of Ex23 skipping was observed for both chemistries, although interestingly was not totally ablated. This is to be expected as the *T*_m_ for the remaining complementary bases is high enough to remain active. Even if the invZEN20 retains some activity, the relative reduction in activity appears similar for both chemistries compared to their parent sequences (**Figure 3a**). Although other nucleotide analogs such as LNA dramatically improve SSO activity, mismatch discrimination of LNA-containing SSOs is severely compromised as compared to regular 2′OMePS RNAs.^28,29^ We directly tested mismatch discrimination of LNA-containing SSOs to ZEN-modified 18-mer SSOs in the HeLa pLuc705 model by inverting four internal nucleotides of the two SSOs (**Figure 3b**). It is immediately noticeable that the LNA18 SSO is not as active as ZEN18 producing less luciferase at all concentrations used. By inverting four central bases in ZEN18, there was a dramatic reduction in activity. In contrast, LNA SSOs showed no significant difference in splice modification at 50 and 25 nmol/l concentrations between the initial 18-mer sequence and inverted sequence. Hence, in accordance with the recent report on antagomirs,^15^ we conclude that incorporation of ZEN raises potency without significantly compromising specificity.

Before testing in *mdx* mice, we assessed the toxicity profile in order to exclude that the raise in observed activity emanates from increased membrane perturbation imparted by the hydrophobic ZEN moiety. Membrane integrity was analyzed following treatment with increasing concentrations of SSO in complex with LF2000 using the Sytox Green assay. Similar to 2OMe20, ZEN20 displayed negligible toxicity in the concentration range used in previous transfection experiments (data not shown). To determine whether these treatments were associated with any long-term toxicity effects, cells were assessed using the Wst-1 cell proliferation assay to measure the activity of mitochondrial dehydrogenase. As seen in **Figure 3c**, neither the 2OMe20 nor ZEN20 affected the viability of cells at the highest concentration (200 nmol/l) used in our experiments. These results were corroborated using another cell viability assay, Cell Titer–Glow, which measures the intracellular levels of ATP (data not shown). These results collectively suggest that inclusion of ZEN has negligible effect on the cell viability *in vitro*.

### ZEN-based SSOs promote exon skipping following intramuscular delivery

**Figure 4:**
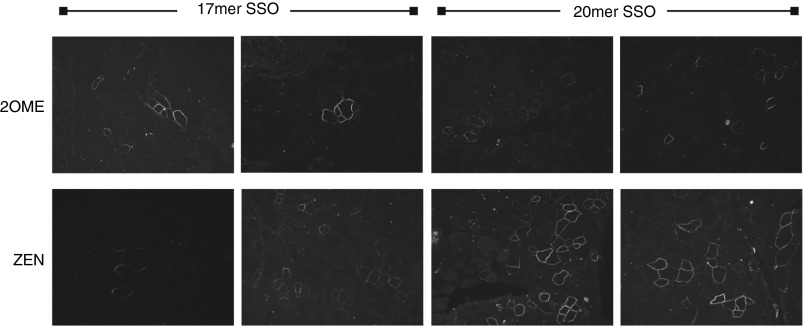
**Intramuscular (IM) administration of 2′OMePS and ZEN-modified 2′OMePS.** Thirty micrograms of 2OMe20, 2OMe17, ZEN20, and ZEN17 SSOs were administered into the tibialis anterior (TA) muscle of *mdx* mouse. Muscles were harvested 2 weeks after administration and sections immunostained for dystrophin to allow quantification of dystrophin-positive fibres. Positive fibre counts were averaged for three animals per treatment group. Dystrophin-positive fibres were as follows: 2OMe20 57 ± 19, ZEN20 71 ± 42, 2OMe17 25.6 ± 8, and ZEN17 49 ± 18 positive fibres. Saline treated TA muscles showed 15 dystrophin-positive fibres.

The promising results *in vitro* led us to assess the *in vivo* potency of ZEN20 and ZEN17. We compared these ZEN-modified SSOs with 2OMe20 and 2OMe17 via intramuscular (IM) injection into the tibialis anterior (TA) of 7–9-week-old *mdx* mice. Two weeks after administration of 30 µg SSO, the TA muscles were harvested and cross sections immunostained for dystrophin positive fibres (**Figure 4**). Separate muscle images were aligned together in order to accurately count all positive fibres, which were spread throughout the muscle section. ZEN20 treatment generated an average of 71 ± 42 dystrophin-positive fibres whereas 2OMe20 generated 57 ± 19 positive fibres. As expected based on the previous *in vitro* results both 17-mers were less active than the 20-mers. However, the difference in activity was more marked between ZEN17 and 2OMe17, generating 49 ± 18 and 25.6 ± 8 dystrophin positive fibres, respectively. Saline-treated TA muscles only showed an average of 15 dystrophin-positive fibres, which represent revertant fibres that are commonly observed in the *mdx* mouse and DMD patients. The muscles were not pretreated to induce damage nor was a delivery carrier used. Although not significant and not with the same magnitude of improvement as in cell culture experiments, exon skipping appeared higher following IM delivery using ZEN.

### ZEN-based SSOs are well-tolerated *in vivo* following systemic delivery

**Figure 5:**
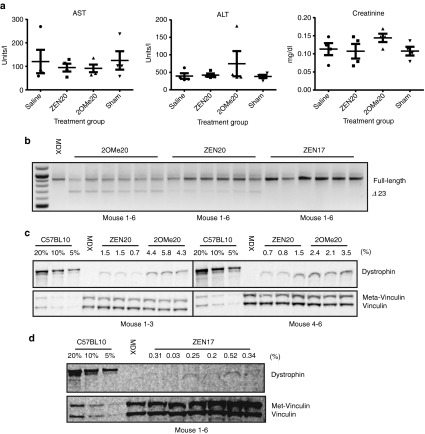
***In vivo* administration of ZEN20 and 2OMe20 splice switching oligonucleotides (SSOs).** (**a**) Safety profile in C57BL/6 mice (*n* = 4) following bolus intravenous (IV) administration of 100 mg/kg SSOs, saline or untreated (sham). 24 hours postadministration, serum was harvested and analyzed for transaminases (aspartate aminotransferase and alanine transaminase) and creatinine. The ZEN modification had no negative effect on the safety profile for liver and kidney function. (**b**) Every second day from 4 to 7 weeks of age, *mdx* mice (*n* = 6) were systemically treated with 100 mg/kg ZEN20, ZEN17, or 2OMe20 for a total of 10 administrations. RT-PCR of RNA isolated from quadriceps harvested either 1 week (ZEN20 and 2OMe20) or 2 weeks postfinal administration (ZEN17). Saline-treated *mdx* and C57BL/10 mice were used as negative and positive controls, respectively. (**c**) Western blot of protein isolated from quadriceps treated with ZEN20 or 2OMe20. Twenty, 10, and 5% total protein loaded for C57BL/10 wild-type animals was used for relative quantification. Quantified dystrophin protein expression is shown above each sample lane as percentage of wild-type levels. 2OMe20 muscle generated an average of 3.75% dystrophin protein as compared to wild-type mice while ZEN20 generated 1.12%, *P* < 0.01(Student's *t*-test). (**d**) Western blot of quadriceps following treatment with ZEN17, generating 0.28% dystrophin protein.

We next evaluated the potential systemic delivery of 20-mer SSOs as they appeared most potent in the IM experiments. Conventional 2′OMePS RNA has been used previously in several systemic *mdx* mouse studies, primarily by Aartsma-Rus and colleagues.^18,20,30^ They have shown that high doses and multiple injections of such SSOs are required to detect dystrophin restoration in skeletal muscle. Based on this, and the fact that ZEN has not previously been tested at high doses *in vivo*, we first performed experiments addressing the acute safety profile of these SSOs. C57BL/6 mice were treated with a bolus injection of either 100 mg/kg of 2OMe20, ZEN20 or saline, or noninjected and examined 24 hours postinjection for clinical toxicology and immunology markers. As seen in **Figure 5a**, none of the treatments altered the levels of transaminases (AST and ALT) or serum creatinine, suggesting negligible effects on liver or kidney function, respectively. Furthermore, markers of inflammation (C-reactive protein and TNFα) were below the detection limit in all groups (data not shown). We then evaluated chronic toxicity of the 20-mer SSOs following 10 IV administrations of 100 mg/kg given every second day to *mdx* mice. Serum extracted upon harvest was assessed for safety profiles after multiple administrations (**Supplementary Figure S3**). No significant alteration of transaminase levels (AST and ALT), alkaline phosphatase (ALP), or serum creatinine was observed. These results suggest that the tested SSOs are safe to use at the tested dose.

### Systemic delivery of SSOs in *mdx* mice reveals important discrepancies in activity

Encouraged by the IM administration results and the systemic safety profile, we initiated a systemic delivery study using naked IV administration. Every second day from 4 to 7 weeks of age, *mdx* mice were treated with 100 mg/kg ZEN20 or 2OMe20 via tail vein systemic administrations for a total of 10 administrations. Saline administered *mdx* and C57BL/10 wild-type animals were used as controls. One week following the final administration, diaphragm, quadriceps, TA, and heart were harvested and dystrophin protein and RNA expression levels assessed. Overall, the levels of dystrophin protein restoration were low in both treatment groups. The greatest level of exon skipping and protein production was observed in the quadriceps (**Figure 5b**,**c**) although a faint band for Ex23-skipped mRNA and dystrophin protein was noted in diaphragm and TA muscles (**Supplementary Figure S1**). No dystrophin protein expression was observed in heart with either of the chemistries (data not shown). Unexpectedly, and in contrast to the IM and *in vitro* lipofection results, 2OMe20 generated a statistically significant increase in dystrophin protein within quadriceps muscle with an average of 3.75% dystrophin protein compared to only 1.12% generated by ZEN20 (*P* < 0.01). The difference in these results is not due to stability of ZEN modified 2′OMe ONs. 2′OMe ONs with and without ZEN modification remain largely intact for 96 hours in 10% fetal bovine serum with the loss of only the end 3′ nucleotide after a few hours. In liver extract, representing intracellular degradation, the ONs are even more stable.^15^ The lower *in vivo* efficacy could be due to the larger molecular weight of ZEN20 (7708 g/mol) over 2OMe20 (6887.5 g/mol), as suggested in previous reports where short 2′-modified chimeric gapmer antisense oligonucleotides (ASOs) showed improved uptake following naked IV administration in mice.^31^ These mice were further assessed through open field behavioural monitoring in the week following final administration.^32,33^ *Mdx* mice treated with ZEN20 and 2OMe20 showed slight improvement in overall activity compared to saline treated *mdx* mice (**Supplementary Figure S2**). Furthermore, there was no change to creatine kinase, reflecting the limited tissue restoration of dystrophin protein (**Supplementary Figure S3d**).

We sought to determine if indeed the differences in molecular weights of ZEN20 and 2OMe20 accounted for the *in vivo* results. The previously tested ZEN17 has a lower molecular weight, 6733 g/mol, than the 2OMe20 and yet was active both *in vitro* and via IM injection route (**Figures 2** and **4**). Therefore, we replicated the dosing scheme for the previous study, injecting *mdx* mice with 100 mg/kg ZEN17 over the course of 3 weeks but harvesting 2 weeks after final administration. Again, dystrophin protein was only notable in quadriceps, albeit at a lower level than 2OMe20 and Zen20 with an average of 0.28% expression (**Figure 5d**).

### Naked uptake of SSOs correlates with *in vivo* activity

Based on the *in vivo* results, we hypothesized that while the ZEN modification can enhance pre-mRNA binding once the SSO has reached the nucleus, it does not improve and indeed may hinder the uptake to this cellular compartment without the aid of a transfection reagent. To test this hypothesis, we assessed the biological activity of SSOs *in vitro* upon transfection in the absence of delivery agent, so-called “gymnotic” delivery. It has been previously reported that “naked” gapmer ONs at high concentrations can be taken up freely by cells and induce biological responses over time.^34,35,36^ This gymnotic uptake is proposed to be dependent on both the molecular weight and the chemical nature of ONs and as such this mode of *in vitro* delivery could more accurately model *in vivo* conditions for tissue uptake.

**Figure 6:**
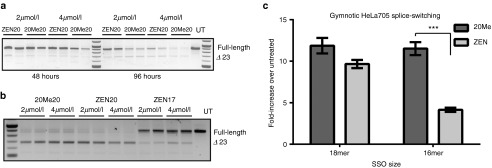
**Naked “gymnotic” transfection *in vitro* correlates with *in vivo* activity.** (**a**) Differentiated H2K *mdx* cells were treated with 2 or 4 µmol/l ZEN20 and 2OMe20 SSOs and harvested 48 (left panel) or 96 hours (right panel) post-treatment. Reverse transcription-polymerase chain reaction (RT-PCR) analysis shows that both SSOs are capable of promoting low levels of exon skipping at 48 hours and higher levels by 96. UT, untreated. (**b**) Gymnotic uptake is greater in dividing cells. Treatment of H2K *mdx* myoblasts seeded at lower confluency with SSOs display much higher activity than in myotubes, although similar between 2OMe20 and ZEN20 at both 2 and 4 µmol/l concentrations. (**c**) Luciferase measurements following gymnotic treatment of pLuc705 HeLa cells with 8 µmol/l of 18-mer and 16-mer 2′OMePS RNA ± ZEN. Three days post-treatment, luciferase activity was analyzed. ***P ≤ 0.0005 (Student's *t*-tests).

In order to delineate the underlying mechanism for the observed discrepancies between *in vitro* and *in vivo* activity for the tested SSOs, we performed naked uptake experiments *in vitro*. In contrast to conventional gymnosis experiments in which ONs are added to cells in a reverse transfection manner (*i.e.*, plating- the cells together with the SSOs) or to cells grown at low confluency, we seeded the cells prior to adding the SSOs in order to more closely resemble *in vivo* conditions. In initial experiments, *mdx* H2K myoblasts were seeded at 80% confluency and allowed to differentiate for two days to reach full confluence. SSOs were subsequently added to cells and left for 48 or 96 hours using either 2 or 4 µmol/l ZEN20 or 2OMe20. According to reverse transcription- polymerase chain reaction (RT-PCR) analysis, both SSOs promote low levels of exon skipping at 48 hours at both concentrations (**Figure 6a**, left panel). By 96 hours, the levels of Ex23-skipped transcript are even greater relative to the full-length band (**Figure 6a**, right panel). In general, the relative efficiency of 2OMe20 and ZEN20 *in vivo* coincides with the gymnotic results. A significant correlation of the *in vivo* response and *in vitro* gymnotic delivery was found using 2 µmol/l at 96 hours (Pearson, *P* = 0.022). In contrast to the previous lipofection results, ZEN20 did not promote higher levels of exon skipping compared to 2OMe20. In fact, the activity of the ZEN20 ON appeared to be slightly lower. These results were corroborated in follow up experiments in myoblasts seeded at lower confluency where both chemistries performed equally well inducing nearly complete exon skipping in these dividing cells (**Figure 6b**). This pattern of activity was also replicated in fully differentiated *mdx* H2K myotube cultures (*i.e.*, nondividing cells) where both ZEN20 and 2OMe20 SSOs displayed similarly low levels of splice switching at 4 days postadministration (data not shown). Thus, it appears that in contrast to cationic transfection, use of the ZEN modifier does not enhance SSO activity in gymnosis experiments. To exclude the possibility that these observations only hold true for the *mdx* myoblast cells, we conducted the same comparative experiment in the previously described HeLa pLuc 705 cells. As seen in **Figure 6c**, after 3 days treatment with 8 µmol/l of HeLa705 ZEN18, levels of luciferase induction were similar to the nonmodified HeLa705 2OMe18. These results contrast with the lipofection data where significantly higher activity was observed with the 18-mer ZEN-modified SSO (**Figure 1c**).

The observation that *in vitro* gymnotic delivery is a better predictor of success *in vivo* than lipofection been previously described using LNA based gapmer ONs.^35^ To compare this data with our own observations of ZEN-based ONs we performed independent experiments using gapmer RNase H active antisense ONs targeting hypoxanthine phosphorybosyltransferase (*HPRT*) mRNA. *HPRT1* was used as a target gene due the long half-life of the protein, allowing silencing experiments to be performed without altering the viability of cells. HeLa cells reverse transfected with 3-10-3 LNA gapmer ON ± ZEN using Lipofectamine RNAiMax, displayed a dose-dependent reduction in *HPRT* transcript levels measured by RT-qPCR. Strikingly, the ZEN-modified gapmer was dramatically more effective at low concentration (3 nmol/l) than the parent gapmer without ZEN (**Supplementary Figure S4a**). In keeping with the SSO results, the improved activity of transfected ZEN modified gapmers was lost under gymnotic delivery conditions (**Supplementary Figure S4b**).

## Discussion

A significant number of diseases are caused by base-pair substitutions or deletions that result in altered splicing patterns.^1^ Many of these are treatable through SSOs, which can directly target the splicing error or manipulate endogenous splicing to by-pass the disease-causing mutation.^37^ Currently, the potential for SSOs as therapeutic agents is still under investigation and SSOs have demonstrated only a modest benefit in clinical trials to date.^13,14^ We have investigated a new non-base modifier, ZEN, for SSOs as a new tool for *in vitro* and potentially *in vivo* application.

ZEN can be incorporated into both 2′OMe and LNA ON chemistries to increase target-binding affinity and thereby improve potency over unmodified ONs. Upon cationic lipid transfection *in vitro*, the enhanced binding affinity of the ZEN modification demonstrated significantly enhanced activity over parent nonmodified SSO in two separate models of splice switching. The increased potency of ZEN modification has been determined to be a result of higher *T*_m_, not improved interaction with the lipid carrier.^15^ Furthermore, it allowed the use of shorter SSOs while maintaining potent activity that was lost with nonmodified equivalent SSOs (**Figure 2c**,**d**). ZEN 2′OMePS SSOs were even more potent than LNA-based SSOs while significantly maintaining better sequence specificity (**Figure 3b**). As such, this makes the ZEN modification a valuable tool for improving the *in vitro* application of anionic ONs.

Given the demonstrated improvement of potency by the ZEN modification *in vitro*, we surmised that this could improve current 2′OMePS SSOs being used in clinical trials for DMD. IM administration of ZEN modified SSOs were modestly more potent than 2′OMePS SSO (**Figure 4**). Disappointingly, this phenomenon was not reproduced *in vivo* when naked IV administration was employed, with the ZEN SSO displaying no advantage to the parent 2′OMePS SSO, although it should be noted that dystrophin restoration was low for both chemistries (**Figure 5**). ZEN modification does not hinder serum stability greatly over 2′OMePS SSOs. Clearance or plasma albumin binding differences were not tested, however, the neutral nature of the ZEN modification does not predict either of these cases. To further understand this result, we assessed gymnotic uptake of these SSOs *in vitro* and observed that the ZEN modification did not confer any advantage, and indeed may have been detrimental to activity compared to the parent SSO. This observation resembles the pattern of activity observed *in vivo*, and led us to conclude that gymnotic transfection is a more robust indicator of SSO activity *in vivo* when naked IV administration is employed.

Discrepancy between *in vitro* and *in vivo* potency has been previously described by Stein *et al.*^35^ In this work, gymnotic delivery of LNA-based gapmers to cultured cells showed comparable potency as systemic *in vivo* administration to the liver.^35^ Similar results were described by Koller *et al*.^34^ who also observed a good correlation between gymnotic uptake in hepatocytes *in vitro* and systemic hepatic delivery in mice of a given anionic ON sequence. However, charge neutral PMO appears to act very differently. Gymnosis transfection in cultured cells requires nearly 10× higher concentration *in vitro* to match 2′OMePS activity yet its *in vivo* activity is much improved in the *mdx* mouse model.^38,39^ Thus, it appears that observations made correlating *in vitro* and *in vivo* potency of anionic ONs for targeting the liver also holds true for skeletal muscle, a difficult tissue to target.

Here, we have explored the benefits of the non-base modifier ZEN as a tool to improve SSO activity in two models of splice switching. This modification appears nontoxic, and can increase binding affinity of an SSO to its target to increase potency. The improved activity was realized when a cationic transfection agent was employed *in vitro* but not observed under naked delivery conditions, either *in vitro* or *in vivo*. Low levels of *in vivo* activity following naked delivery only correlated with *in vitro* activity under gymnotic conditions, suggesting this is a better predictor of naked *in vivo* activity. We therefore consider that while ZEN-modification is an effective tool for *in vitro* work with a cationic transfection agent, translating this improved efficacy to an *in vivo* model will also require an effective delivery agent. Identifying a systemic delivery agent for use *in vivo* is outside the scope of this work, however many groups are working toward this goal.^40,41,42^ Toward this end, John Tyson (University of British Columbia) reports successfully using ZEN-SSOs to alter mRNA splice patterns in targeted transcripts in rat brain using direct CNS injection with facilitated lipid nanoparticle delivery (J Tyson, personal communication, to M.A.B.). Should a successful delivery agent be realized we would anticipate that ZEN-modified ONs will become a useful tool for *in vivo* applications.

## Materials and methods

*Oligonucleotide synthesis.* All ONs employed in this study were synthesized using standard phosphoramidite chemistry and used in sodium salt form (Integrated DNA Technologies, Coralville, IA). The predicted masses for the ONs were verified by electrospray-ionization mass spectrometry and were within ± 0.02%. The ON concentrations were determined from UV absorbance at 260 nm and estimated extinction coefficients. Each ZEN modification increased the ON extinction coefficient by 13,340 l/(mol.cm).

*Cell culture and SSO treatment of cells.* Mouse H2K *mdx* muscle cells^43^ were grown on gelatinized plates at 33 °C, 10% CO_2_ in Dulbecco's modified Eagle's medium with glutamax supplemented with 20% fetal bovine serum, 0.5% Chick Embryo extract, 200 U/ml penicillin, and 200 µg/ml of streptomycin (Invitrogen, Renfrewshire, Scotland). H2K *mdx* myotubes were generated in matrigel-coated 24-well plates by seeding 30,000 cells/well, leaving them for 2 days to reach 90% confluency before changing to starvation media (Dulbecco's modified Eagle's medium supplemented with 5% horse serum) and transferring them to 37 °C, 5% CO_2_ incubator for another 4 days. Cells were transfected using Lipofectamine 2000 (LF2000) (Invitrogen) according to manufacturer's protocol. Briefly, 2.2 µl of LF2000 was used per microgram of SSO. Complexes were formed in 50 µl opti-MEM (Invitrogen) and added to cells grown in 450 µl full growth media. Cells were processed 48 hours later in all transfection experiments. For gymnosis experiments, myoblasts or myotubes were treated using 2 or 4 µmol/l SSO in 500 µl opti-MEM and analyzed for exon skipping 48 or 96 hours later.

HeLa pLuc705 cells were grown at 37 °C, 10% CO_2_ in Dulbecco's modified Eagle's medium with glutamax supplemented with 200 U/ml penicillin and 200 µg/ml of streptomycin (Invitrogen). For transfection experiments, 50,000 cells/well were seeded in 24-well plates and transfected the day after using LF2000 as described above. Cells were lysed using 0.1% Triton X-100 and measured for luciferase expression using Promega Luciferase assay kit (Promega, Southampton, UK) according to manufacturer's instructions on a GloMax (Promega). Gymnosis treatments were conducted as above using 8 µmol/l SSO and assessing luciferase expression 96 hours post-treatment.

*Cell viability assay.* Cell viability was assessed by the Wst-1 proliferation assay according to manufacturer's protocol (Roche Diagnostics Scandinavia AB, Stockholm, Sweden). Briefly, cells were grown and treated as described above but in 96-well format (*i.e.*, 8,000 cells seeded and treated in 100 µl volume). Two days after transfection with 200 nmol/l SSOs complexed with LF2000, Wst-1 was added to each well. Wst-1 measures the activity of mitochondrial dehydrogenases to convert tetrazolium salts to formazan and cell proliferation is directly correlated to the amount of formazan product that is formed. Absorbance was measured on SpectraMax Absorbance Microplate reader (Molecular Devices, Sunnyvale, CA).

*Animals.* Experiments were carried out in the Biomedical Sciences Unit, University of Oxford according to procedures authorized by the UK Home Office. Experiments were performed in *mdx* mice (C57BL/10ScSn-Dmd^mdx^/J) and C57BL/10 and C57BL/6 wild-type controls.^44^

*IM and systemic intravenous injections.* IM injections were carried out on 7–9-week-old *mdx* mice under general anaesthesia. Thirty micrograms of ON in 30 µl volume of 0.9% saline were injected into each TA. Intravenous (IV) administration was administered via tail vein in restrained *mdx* and C57BL/10 wild-type female mice from 4 to 7 weeks of age. ONs were prepared in 0.9% saline solution at a final dose of 100 mg/kg. Animals were sacrificed by rising CO_2_ inhalation and tissues were harvested 1 (20-mer IV injections) or 2 weeks (20-mer and 17-mer IM and 17-mer IV injections) postadministration. Skeletal muscle and heart were snap-frozen in isopentane, cooled in dry ice, and stored at −80 °C.

*RNA extraction and RT-PCR.* Total RNA was extracted from cells as well as control and treated mouse tissues using TRIzol reagent (Invitrogen, Carlsbad, CA) following manufacturer's instructions. Four hundred nanograms of RNA template was used in a 50 µl reverse transcription reaction using One Step RT-PCR kit (Qiagen, Hilden, Germany) and gene-specific primers (Ex 20–26, Fwd: 5′-CAG AAT TCT GCC AAT TGC TGA G-3′, Rev: 5′-TTC TTC AGC TTG TGT CAT CC-3′). Cycle conditions: 50 °C for 30 minutes, followed by 30 cycles of 30 seconds at 94 °C, 1 minute at 58 °C, and 2 minutes at 72 °C. Two microliters of cDNA was further amplified in a 50 μl nested PCR (QIAGEN PCR kit) using the following cycle conditions: 94 °C for 30 seconds, 58 °C for 1 minute, and 72 °C for 1 minute for 24 cycles (Ex 20–26: Fwd: CCC AGT CTA CCA CCC TAT CAG AGC, Rev: CCT GCC TTT AAG GCT TCC TT). PCR products were examined by electrophoresis on a 2% agarose gel.

*Protein extraction and western blot.* Control and treated muscle samples were homogenized in lysis buffer comprising 75 mmol/l Tris–HCl (pH 6.5) and 10% sodium dodecyl sulphate complemented with 5% 2-mercaptoethanol. Samples were heated at 100 °C for 3 minutes before centrifugation and supernatant was saved. Protein levels were measured by Bradford assay (Sigma, St Louis, MO) and quantified using BSA standards. Ten to 15 µg of protein of saline and SSO treated *mdx* sample, and 20, 10, and 5% of these concentrations of C57BL10 protein (positive control) were loaded onto 3–8% Tris-Acetate gels. Proteins were blotted onto polyvinylidene fluoride membrane and probed for dystrophin using DYS1 (Novocastra, Buffalo Grove, IL) and loading control, α-actinin (Sigma), antibodies. Primary antibody was detected by binding of IRDye 800CW goat-anti mouse IgG (LI-COR). Western blots were imaged (LiCOR Biosciences, Lincoln, NE) and analyzed using the Odyssey imaging system.

*Immunohistochemistry and histology.* Transverse sections of tissue samples were cut at 8 µm thickness for visualization of dystrophin expression. Sections were costained with rabbit-anti-dystrophin (Abcam, Cambridge, MA) and rat anti-laminin (Sigma), and detected by goat-anti-rabbit immunoglobulin G Alexa 594 and goat-anti-rat immunoglobulin G 488 secondary antibodies respectively (Invitrogen). Images were captured using a Leica DM IRB microscope and Axiovision software (Carl Zeiss, Cambridge, UK).

*Clinical biochemistry.* Acute safety profile was analyzed in C57BL/6 wild-type mice given a bolus injection of 100 mg/kg ONs in 0.9% saline solution. Serum was taken from the mice by cardiac puncture. Analysis of serum alanine transaminase (ALT), aspartate transaminase (AST), C-reactive protein (CRP), and creatinine levels was carried out by the clinical laboratory at Karolinska University Hospital Huddinge, Stockholm Sweden by standard methods. The TNF-α levels were measured by enzyme-linked immunosorbent assay. A precoated enzyme-linked immunosorbent assay-kit designed for mouse TNF-α was purchased from BioLegends (San Diego, CA) and the samples were analyzed according to the manufacturer's instructions. Safety profile following multiple high-dose administration was determined through serum samples taken from the jugular vein of *mdx* mice immediately following sacrifice by CO_2_ inhalation. Each animal had received ten administrations of 100 mg/kg ZEN20 or 2OMe20 in 0.9% saline solution. Analysis of toxicity biomarkers was performed by clinical pathology laboratory, Mary Lyon Centre, MRC, Harwell, UK.

*Open field animal activity monitoring.* Behavioural and locomotor measurements were recorded using the Linton AM1053 X, Y, Z IR Activity Monitors. Mice were acclimatized 1 hour daily for 3 consecutive days beginning the day after the final systemic IV administrations. Data collection was performed in the same cages used for acclimatization over a 90-minute period for 5 consecutive days. The first 30 minutes of data for each day was discarded at the end of each trial. Twenty-two different activity parameters were measured: activity (total, slow, and fast), static counts (total, slow, and fast), mobile counts (total, slow, and fast), rearing counts (total, slow, and fast), centre rearing counts (total, slow, and fast), active time, static time, mobile time, rearing time, front to back counts, inactive time, and distance travelled.

*Statistical analysis.* Principal component analysis from a correlation matrix was calculated on the 22 activity parameters corresponding to the open field study using the software SPSS (SPSS, Chicago, IL). The components explaining the highest percentage of the variance were displayed on two-dimensional plots. Student's *t*-test and Pearson correlations were performed in R (http://www.r-project.org/).

**SUPPLEMENTARY MATERIAL** **Figure S1.** Dystrophin expression in TA and diaphragm following in vivo administration of ZEN20 and 2OMe20 SSOs. **Figure S2.** Changes to behaviour and activity following treatment with ZEN20 and 2OMe20. **Figure S3.** Serum safety profile of mdx mice treated with ZEN20, 2OMePS20 or saline. **Figure S4.** Effect of the ZEN-modifier on RNase H active antisense ASO knockdown varies between lipofection and gymnotic delivery.

## Figures and Tables

**Table 1:**
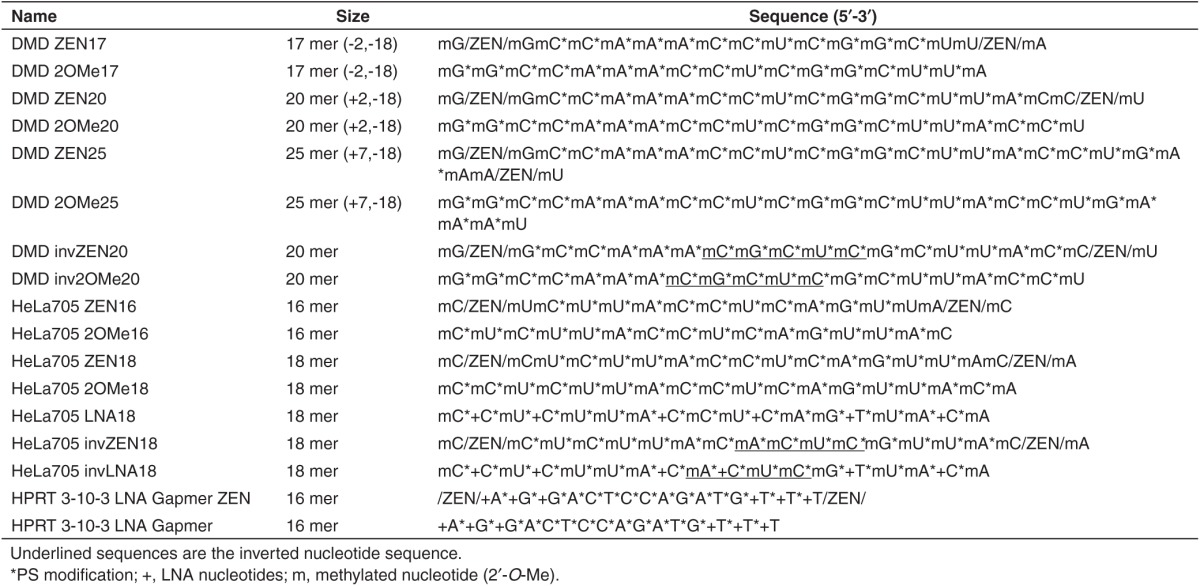
Nomenclature, size, and sequence of oligonucleotides

## Supplementary Material

Supplementary Figure S1 Dystrophin expression in TA and diaphragm following in vivo administration of ZEN20 and 2OMe20 SSOs.

Supplementary Figure S2 Changes to behaviour and activity following treatment with ZEN20 and 2OMe20.

Supplementary Figure S3 Serum safety profile of mdx mice treated with ZEN20, 2OMePS20 or saline.

Supplementary Figure S4 Effect of the ZEN-modifier on RNase H active antisense ASO knockdown varies between lipofection and gymnotic delivery.
