# Supplementary figures and images for: Correlating In Vitro Splice Switching Activity With Systemic In Vivo Delivery Using Novel ZEN-modified Oligonucleotides
Source: Mol Ther Nucleic Acids. 2014 Nov 25;3(11):e212–. doi: 10.1038/mtna.2014.63 (PMC4459549; doi:10.1038/mtna.2014.63)

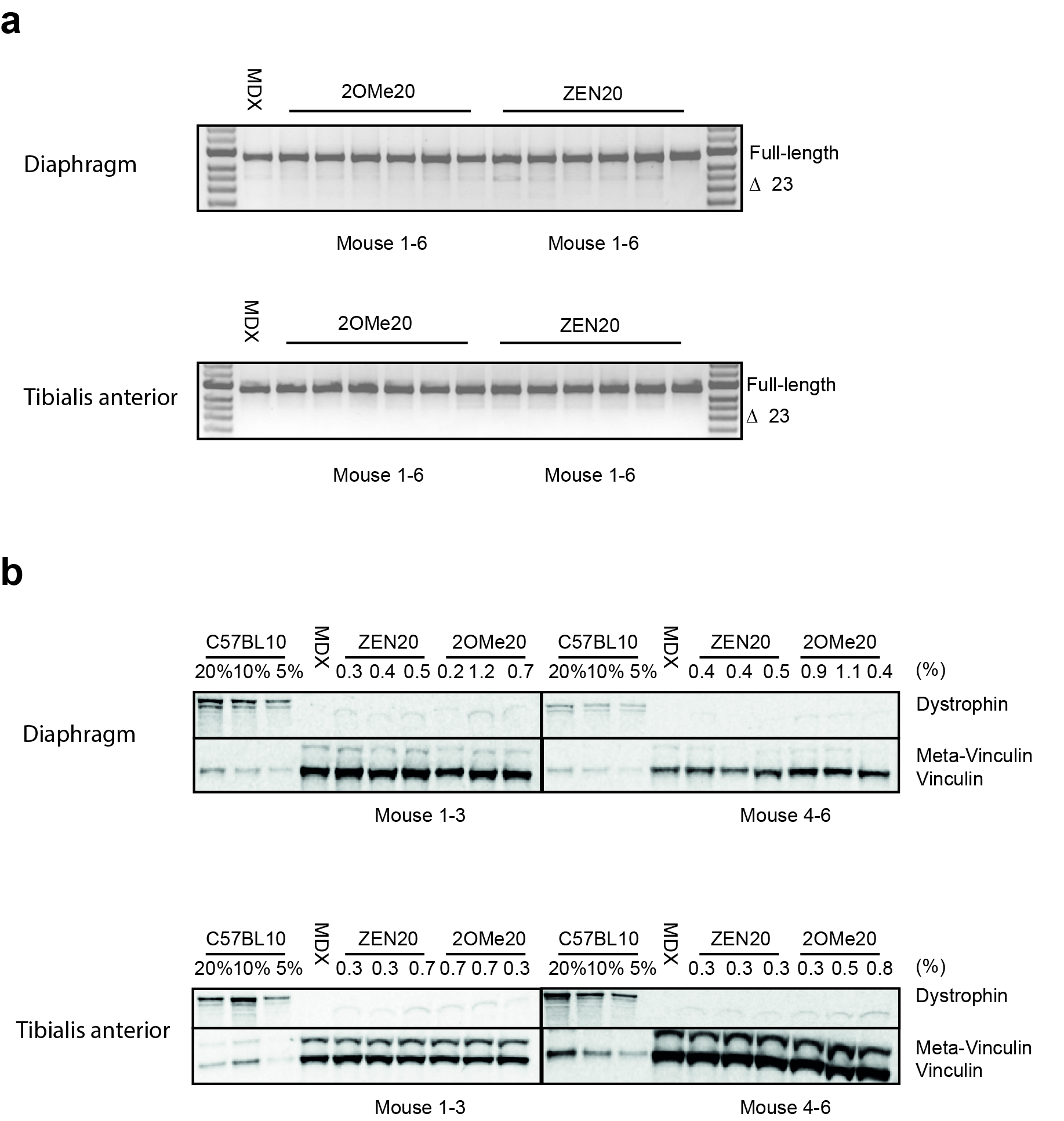

Supplement: Supplementary Figure S1 — Dystrophin expression in TA and diaphragm following in vivo administration of ZEN20 and 2OMe20 SSOs. [file mtna201463x1.tiff]

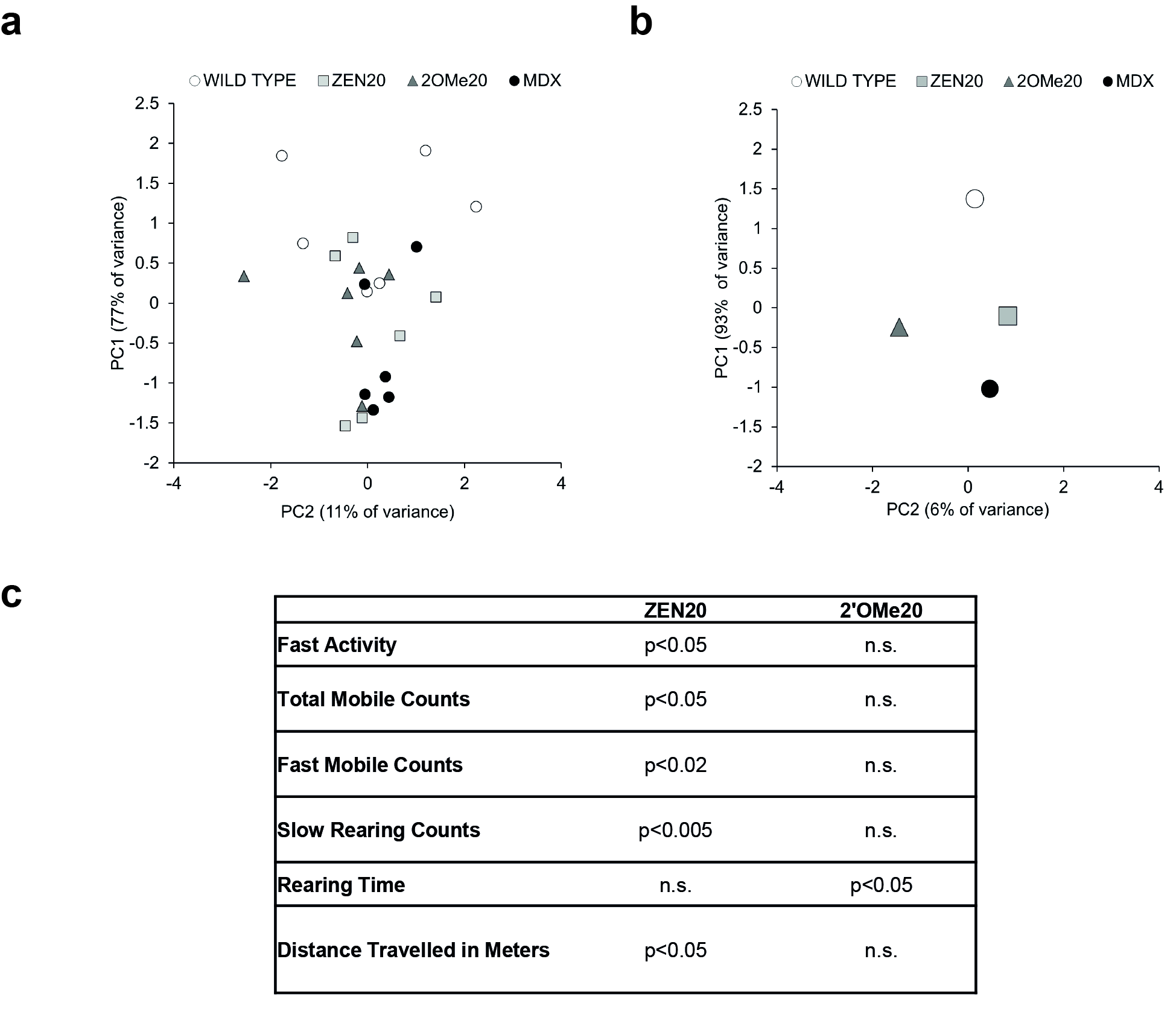

Supplement: Supplementary Figure S2 — Changes to behaviour and activity following treatment with ZEN20 and 2OMe20. [file mtna201463x2.tiff]

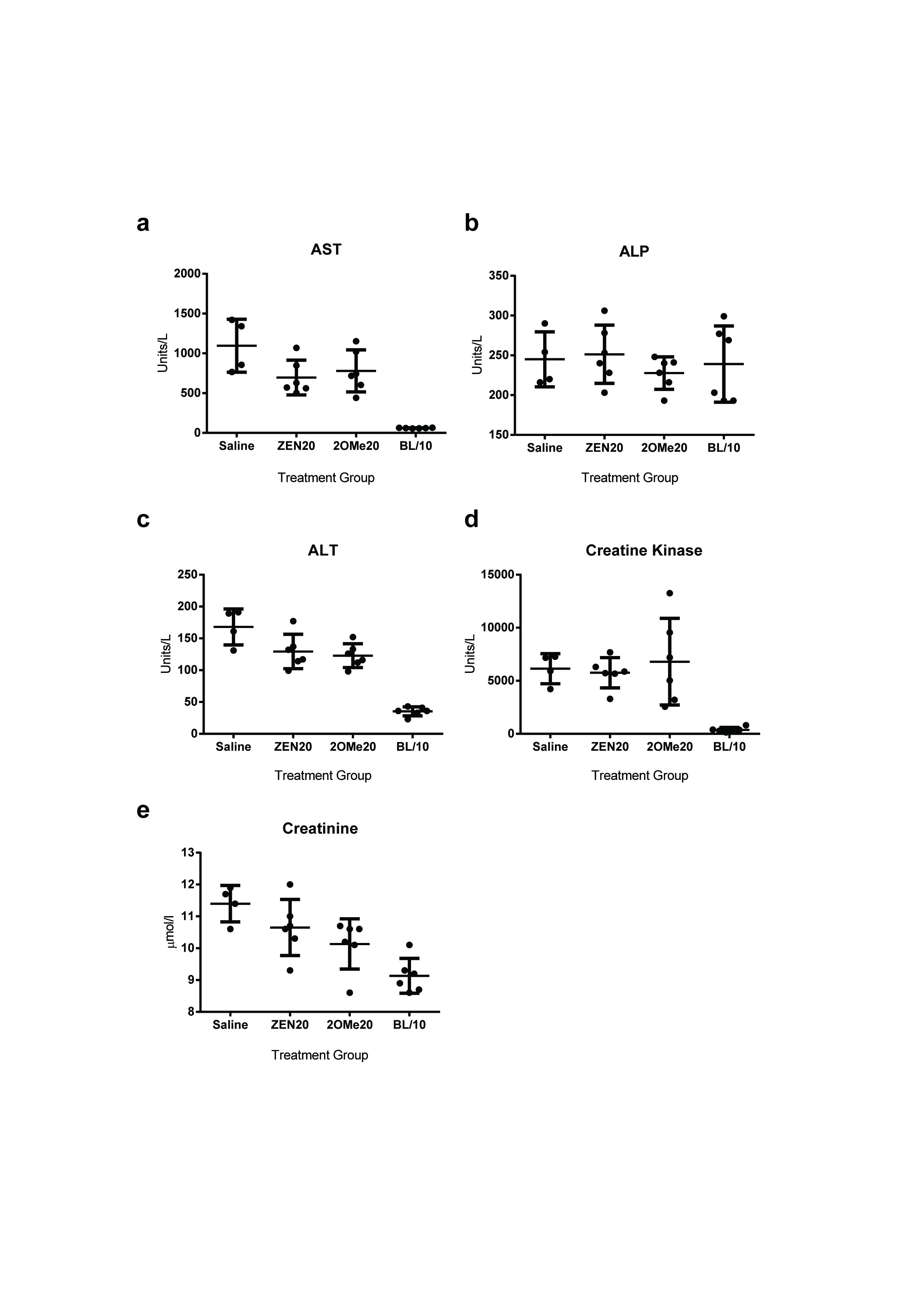

Supplement: Supplementary Figure S3 — Serum safety profile of mdx mice treated with ZEN20, 2OMePS20 or saline. [file mtna201463x3.tiff]

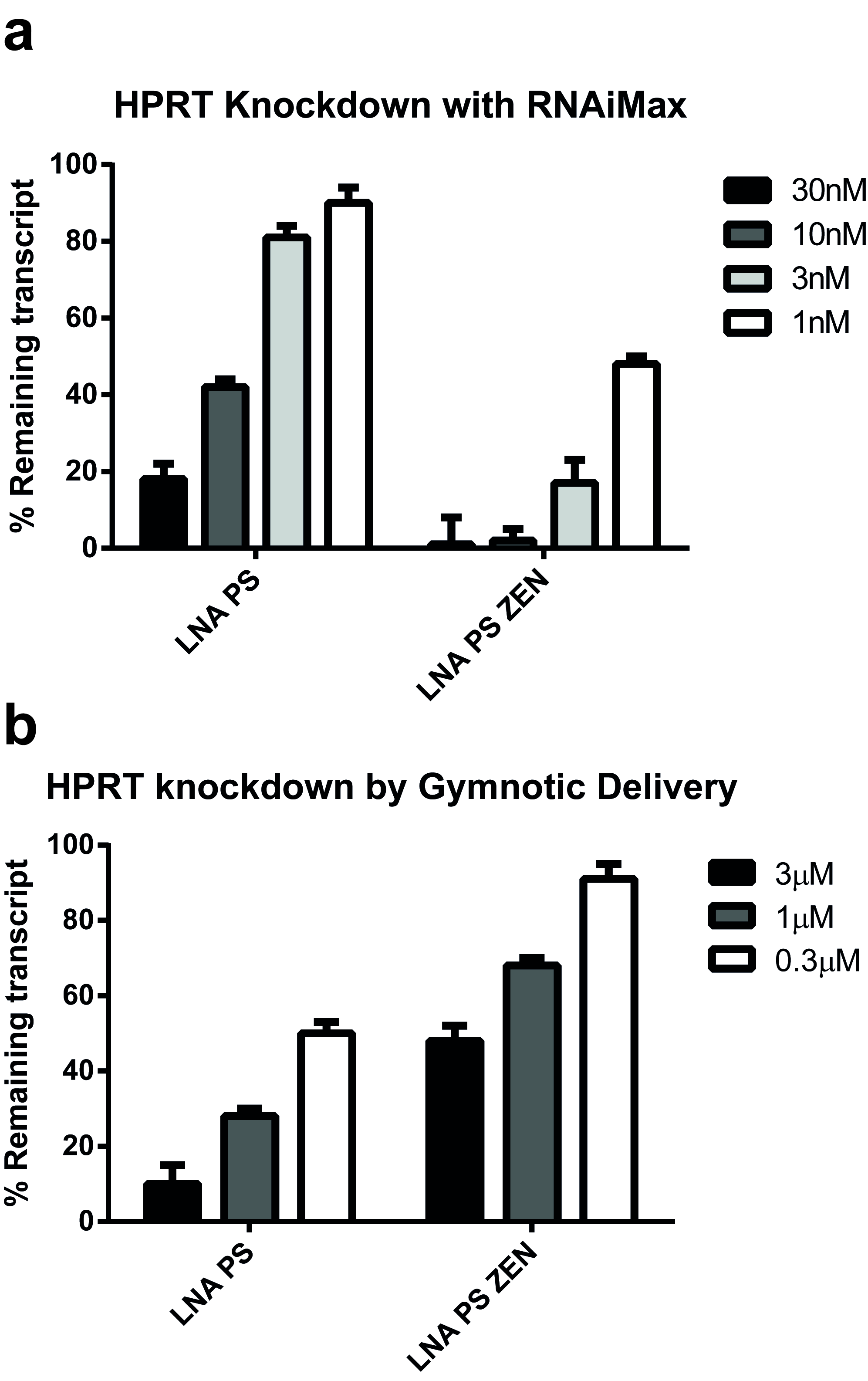

Supplement: Supplementary Figure S4 — Effect of the ZEN-modifier on RNase H active antisense ASO knockdown varies between lipofection and gymnotic delivery. [file mtna201463x4.tiff]
